# Rethinking SME default prediction: a systematic literature review and future perspectives

**DOI:** 10.1007/s11192-020-03856-0

**Published:** 2021-01-29

**Authors:** Francesco Ciampi, Alessandro Giannozzi, Giacomo Marzi, Edward I. Altman

**Affiliations:** 1 University of Florence, Via delle Pandette, 9, 50127 Florence, IT Italy; 2 University of Lincoln, Brayford Pool, Lincoln, GB LN6 7TS UK; 3 NYU Salomon Center, Leonard N. Stern School of Business, New York University, 44 West 4th Street, New York, NY 10012 USA

**Keywords:** Default prediction, SMEs, Credit risk, Risk prediction, Bankruptcy, Systematic literature review, Bibliometric analysis, VOSviewer, Credit scoring, Rating, SME survival, Failure

## Abstract

Over the last dozen years, the topic of small and medium enterprise (SME) default prediction has developed into a relevant research domain that has grown for important reasons exponentially across multiple disciplines, including finance, management, accounting, and statistics. Motivated by the enormous toll on SMEs caused by the 2007–2009 global financial crisis as well as the recent COVID-19 crisis and the consequent need to develop new SME default predictors, this paper provides a systematic literature review, based on a statistical, bibliometric analysis, of over 100 peer-reviewed articles published on SME default prediction modelling over a 34-year period, 1986 to 2019. We identified, analysed and reviewed five streams of research and suggest a set of future research avenues to help scholars and practitioners address the new challenges and emerging issues in a changing economic environment. The research agenda proposes some new innovative approaches to capture and exploit new data sources using modern analytical techniques, like artificial intelligence, machine learning, and macro-data inputs, with the aim of providing enhanced predictive results.

## Introduction

Effective default risk prediction of small and medium enterprises (SMEs) has always been a concern of financial institutions and bank managers, attracting the interest of academics from the 1970s (Edmister 1972; Laitinen 1993).

Starting from the 1990s, the topic acquired renewed attention as a result of the implementation of the Basel Capital Accords, which had a significant impact on loan pricing and credit risk management processes. With the adoption of Basel II in 2004, banks were forced to compute their capital requirements based on the ratings assigned to their borrowers by their internal rating systems, including SMEs. Furthermore, accuracy rates of traditional default prediction models, which had been based mainly on samples of large firms, has proved to be quite low when applied to SMEs, which have their unique financial characteristics (Ciampi 2015). The combination of these factors has led banks, non-bank lenders and FinTech service firms, as well as academics, to focus their attention on the analysis of SME default prediction as a specific and autonomous field of study.

In addition to the impetus from Basel II, during the 2007 to 2009 global financial crisis, SMEs faced increased difficulties in obtaining credit, and policymakers, as well as lenders**,** were highly committed to managing the effects of the progressive increase of SME defaulted loans. As a consequence, the need to have better default prediction models, especially for SMEs, dramatically increased and returned to the heart of the academic and managerial debate (Oliveira et al. 2017).

This need has been made all the more crucial by the global effects of the COVID-19 pandemic, which, while having a great impact on companies of all sizes, proved to affect SMEs to a larger extent because of their physiologic financial weakness (Ciampi 2015), as well as their prevalence in the industries and countries more exposed to the effects of the pandemic (Adian et al. 2020). Using a large firm level data set, Orbis, from BvD-Moody’s, Gourinchas et al. (2020) estimate that, without access to governmental liquidity measures, under COVID-19 the default rate for SMEs in seventeen countries will increase by nearly 9 percentage points in 2020. A recent a survey of more than 5,800 small firms between March 28 and April 4, 2020 conducted by Bartik et al. (2020) find many small businesses are financially fragile with the median business having more than $10,000 in monthly expenses and only about two weeks of cash on hand at the time of the survey. This extremely challenging and dramatic economic context is making the limits of traditional rating models even more pronunciated when applied to SME default prediction. These models are mainly based on financial ratios and accounting data and their SME default prediction accuracy is therefore historically low (Ciampi and Gordini 2013). This aspect is being further called into question by the effects of the COVID-19 global crisis that are strongly impacting the financial health of the vast majority of SMEs and forcing them to base their chances of survival on turnaround plans which, by their very nature, represent a sharp break with the past, thus significantly reducing the predictive value of past accounting data on which financial ratios are based. Furthermore, this crisis is expected to have an amplification effect on the tendency of SMEs to resort to unorthodox accounting behaviors with the aim of postponing the emerging of their economic and/or financial imbalances (Ciampi 2018), thereby enlarging the time within which a firm financial weakness is reflected by its financial ratios level. This fact is particularly critical considering that the information provided by SMEs is physiologically more opaque than that provided by larger firms, which makes the issue of evaluating their creditworthiness a difficult one (Ciampi 2017; Duarte et al. 2018; Frame and Woosley 2004).

Generally, the specific features of SMEs make it necessary to build default prediction models tailored around SMEs’ specific issues and based on qualitative information in addition to traditional financial ratios (Ciampi 2015, 2018; Laitinen and Gin Chong 1999; Norden and Weber 2010). In this connection, over the last dozen years, the studies around SME default prediction have evolved into a relevant research domain by addressing several potentially new issues and techniques (Ciampi 2015). The predictive power of several non-financial variables, in addition to the traditional financial ratios, has been tested by scholars coming from different fields such as finance, banking, management, statistics, and operational research. This combination of concurrent use of non-traditional quantitative methodologies and non-financial predictive variables has allowed improvements in the prediction accuracy of SME models over the years (Ciampi and Gordini 2013). Nevertheless, the continued presence of significant classification errors in the prediction models proposed in the literature highlights the need to increase the types of predictors, as well as to analyse a higher number of non-financial, qualitative predictors. Furthermore, the most advanced studies in the field are able to jointly consider only a few predictive variables within the same model and a prediction system based on a consistent number of different variables is still missing^1^ (Ciampi 2015).

Internal rating models should be modified and enhanced according to the exceptional economic conditions which have been generated by the 2007–2009 global financial crisis and the COVID-19 crisis and are less and less reflected in historical accounting data traditionally used to build the rating systems. Default prediction models for SMEs should be calibrated and validated with relevant changes in the predictive variables, or changes in the weightings of the variables, e.g. by increasing the role of future oriented, qualitative variables in a scenario of significant, unavoidable, deterioration of financial ratios for the majority of SMEs.

Similar changes should be done for the ongoing credit monitoring with the need to specify which new indicator changes should determine rating downgrades and to consider the subjectivity of credit analysts able to adequately evaluate the value-added soft information when assessing SMEs creditworthiness in the current situation.

We believe that in the post-COVID-19 world the ability to survive for SMEs will increasingly depend on their innovation skills, adaptability to new realities and human and relationship capital. As a consequence, banks and new e-based lenders will be forced to rethink the credit risk assessment of SMEs by including in their internal rating models additional qualitative variables related to all these factors.

Despite the clear relevance assumed by this field of research and the debate around the strategic importance of effective SME default prediction, there is yet no systematic literature review about SME default prediction that organises extant articles by integrating contributions from the different managerial and academic perspectives. This paper aims to bridge this gap and to answer the ‘call’ for new predictors imposed by the new and complex challenges we are facing nowadays. We will attempt to do this through the use of bibliometric analysis via a systematic literature review and by proposing several promising future research avenues. These future research avenues may drive scholars and practitioners in designing new approaches to evaluate the credit risk of SMEs in the post-Covid-19 economic environment. In doing so, the paper is structured as follows. After the introduction, Sect. 2 presents in detail the methodology used to conduct the study. Section 3 presents the results of the bibliometric activity indicators, while Sect. 4 presents the VOS (Visualization of Similarities) analysis and the following systematic literature review. Section 5 stresses the main future research avenues which have emerged and proposes several important research questions that need to be addressed. Finally, Sect. 6 presents conclusions and limitations of the present paper.

## Methodology

In order to offer a non-biased and fine-grained analysis of the literature concerning the topic of SME default prediction, we opted for a hybrid approach combining a quantitative bibliometric analysis and a systematic literature review. This approach has already been adopted in other field of studies, such as sustainability (Mura et al. 2018), innovation (Klarin 2019; Marzi et al. 2021), knowledge management (Fakhar Manesh et al. 2021; Pellegrini et al. 2020), strategy (Ciampi et al. 2020) and entrepreneurship (Delgado García et al. 2015; Sassetti et al. 2018), where it showed its effectiveness and reliability (Ding et al. 2014).

For the bibliometric analysis proposed by the present study the VOS analysis was used (van Eck et al. 2006; van Eck and Waltman 2010), while the systematic literature review follows the approach suggested by Tranfield et al. (2003).

First of all, we built our research query. In this connection, we accomplished an exploratory review of the published papers concerning SME default prediction in order to build an updated overview of the topic under study, as well as a list of the terms and keywords mostly used by scholars. After several iterations and refinements (Costa et al. 2017; Ding et al. 2014; Fakhar Manesh et al. 2021; Zupic and Čater 2015), we obtained the following research query that enabled us to effectively retrieve all the significant papers published on the topic under study and present in the major scientific databases: (sme OR smes OR "small enterprise*" OR "small and medium size enterprise*" OR "small compan*" OR "small business*") AND (default OR bankruptcy OR failure OR "credit risk*" OR "financial distress") AND ( prediction OR predicting OR "credit risk*") AND (scoring OR rating).

We ran the query in Scopus using the operator “TITLE-ABS-KEY”. We also ran the same query in Web of Science with the operator “TS”. The aforementioned operators perform research in titles, abstracts and keywords inside each document indexed in the database. We limited our query to “articles” and “literature reviews” (Delgado García et al. 2015; Pellegrini et al. 2020; Sassetti et al. 2018) and excluded subject areas not pertinent to the scientific domain object of our research.

A cross-validation was also made using the Web of Science Database, the results of which highlighted that Web of Science did not index any additional relevant paper compared to Scopus Database. Furthermore, it did not contain some old but relevant papers which were instead present in the Scopus Database (e.g. Keasey and Watson 1986, 1987; Shailer 1989). Therefore, we opted for Scopus as our primary source of data (Falagas et al. 2008; Marzi et al. 2021). Our initial dataset from Scopus was retrieved on the 17th of February in 2020 and was composed of 354 entries.

The second step was dedicated to define the inclusion criteria to be used in order to select the final list of papers to be included in our literature review. This second step was necessary because in order not to miss any relevant paper we opted for a very inclusive research query. We approached this cleaning process using a combination of inclusion criteria (López-Fernández et al. 2016; Marzi et al. 2017, 2021; Mura et al. 2018; Sassetti et al. 2018) based on (1) the definition of SME, (2) the definition of default, and (3) the objectives of the present research. Regarding the first point, we included in the final dataset only the documents adopting a definition of SME in line with the one from the European Commission: an independent firm with less than 250 employees, a turnover below 50 million euros, and a balance sheet below 43 million euros. Second, according to the prevailing corporate default prediction literature, we used a broad definition of the default event that included not only the initiation of bankruptcy proceedings but also the presence of significant financial difficulties, because of which companies may lose the financing granted (Ciampi 2015). Finally, we selected all the documents: (1) aiming to develop and/or test SME default prediction models and/or default predictors based on both financial and/or non-financial predictive information (Altman et al. 2020; Altman and Sabato 2007; Ciampi 2015); (2) focused on the analysis, development and/or testing of SME credit risk models from both lenders’ and/or borrowers’ perspectives (Glennon and Nigro 2011). At the end of this second step a refined dataset made of 111 documents was obtained.

In the third step, we performed a bibliometric analysis. First, we analysed a series of bibliometric indicators (such as the number of papers per year and the main journals in which the papers have been published) showing the volume and impact of the papers included in our dataset (Fernandez et al. 2015). Subsequently, we moved to the core of our bibliometric analysis by implementing the VOS analysis. The aggregation criterium bibliographic coupling was applied (van Eck et al. 2006; van Eck and Waltman 2010). This kind of coupling exists when two documents, A and B, both cite a third document, C (Zupic and Čater 2015). The more two or more papers are bibliographically coupled, the more they can be supposed to have a similar approach when exploring the topic of interest (van Eck and Waltman 2010; Zupic and Čater 2015). We used bibliographic coupling because of its capacity in answering the following questions: “How does the intellectual structure of the research stream reflect the richness of the theoretical approaches? How has the intellectual structure of a small niche X developed through time?” (Zupic and Čater 2015, p. 439). The VOS analysis allows to develop a matrix of normalised co-occurrences of items, in the present case the references (van Eck and Waltman 2007), and create a map in which the papers are located in the axes *x* and *y* so that their closeness reflects their similarity in term of references. The smaller is the distance between the items; the stronger is the relationship between the items (van Eck and Waltman 2010). It also allows to perform a clusterisation process that links items together in function of their base of shared references (van Eck and Waltman 2010). If items belong to the same cluster, they are strongly related in terms of common references, thus representing a sub-stream of research (van Eck and Waltman 2010). This analysis highlighted that 106 papers, out of the 111 comprised in our refined dataset, were bibliographically coupled. Only five documents were not connected in term**s** of shared references and consequently excluded from the similarity analysis (Angilella and Mazzù 2019; Cornée 2019; Edmister 1972; Laitinen 1993; Liu et al. 2019). Following the suggestion of van Eck and Waltman (2010) we calibrated the VOSviewer routines using the association strength for the normalization process, a resolution value of 1.00, and minimum cluster size value of 1. For presenting the results of our VOS analysis we set the scale to 1.00 and weighted the items using the normalised citations, thus ensuring an adequate representation even for the most recent papers (Ding et al. 2014; van Eck and Waltman 2010). The results of the VOS analysis (see Fig. 2, Sect. 4) highlight a strong nexus of connections, totalling 2,030 links and a total link strength value of 4977.

Finally, in the last step, grounded on the output of the VOS analysis, a systematic literature review was carried out (Tranfield et al. 2003): we systematically analysed all the papers inside each cluster highlighting the main topics, the main findings and proposed a series of research avenues that still need to be further explored by scholars and practitioners (Fakhar Manesh et al. 2021; Klarin 2019; Marzi et al. 2021; Mura et al. 2018). The list of the 106 papers included in the present study is provided in the appendix of the paper.

## Results of the bibliometric activity indicators

**Fig. 1:**
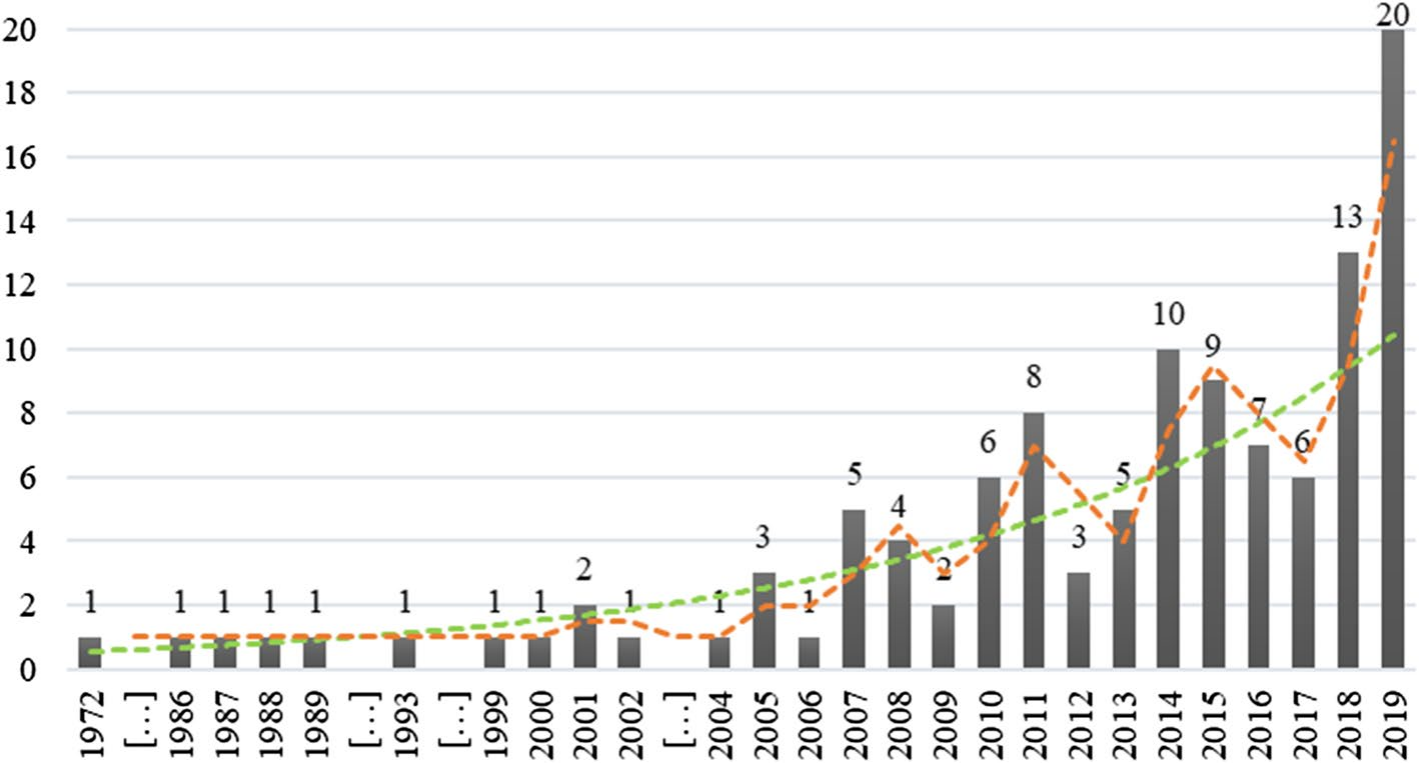
Distribution of the papers over the years

As the first bibliometric activity indicator, Fig. 1 shows how the papers are distributed over time.

The interest in the field started in the early 1970s. However, it was only after 2004, the year of the initial publication of the Basel II Accord, which linked the minimum required levels of the capital of financial institutions to the level of creditworthiness of their clients more strictly, where we recorded a gradual but growing interest by scholars, which reached a peak in the last year (2019) of the period of our analysis. In Table 1, we present the leading journals in which the SME default prediction literature was published (at least two papers).

**Table 1:** Main journals publishing studies on default prediction

Journal	Number of papers
Journal of small business management	11
Journal of the operational research society	9
Journal of banking and finance	7
Journal of financial services research	6
Small business economics	6
Accounting and business research	3
Journal of financial intermediation	3
Journal of small business and enterprise development	3
Management decision	3
Applied financial economics	2
Competitiveness review	2
Economic modelling	2
Emerging markets finance and trade	2
International journal of entrepreneurship and small business	2
International review of financial analysis	2
Journal of money, credit and banking	2
Journal of risk finance	2
Managerial finance	2
Review of quantitative finance and accounting	2
Studies in economics and finance	2

These journals are, not surprisingly, centered around the field of small business management and finance. The only exception is the *Journal of the Operational Research Society,* which published several papers illustrating applications of operational research techniques to the prediction of SME default and the lenders’ decision about loan approval.

## Results of the VOS analysis and the systematic literature review

**Fig. 2:**
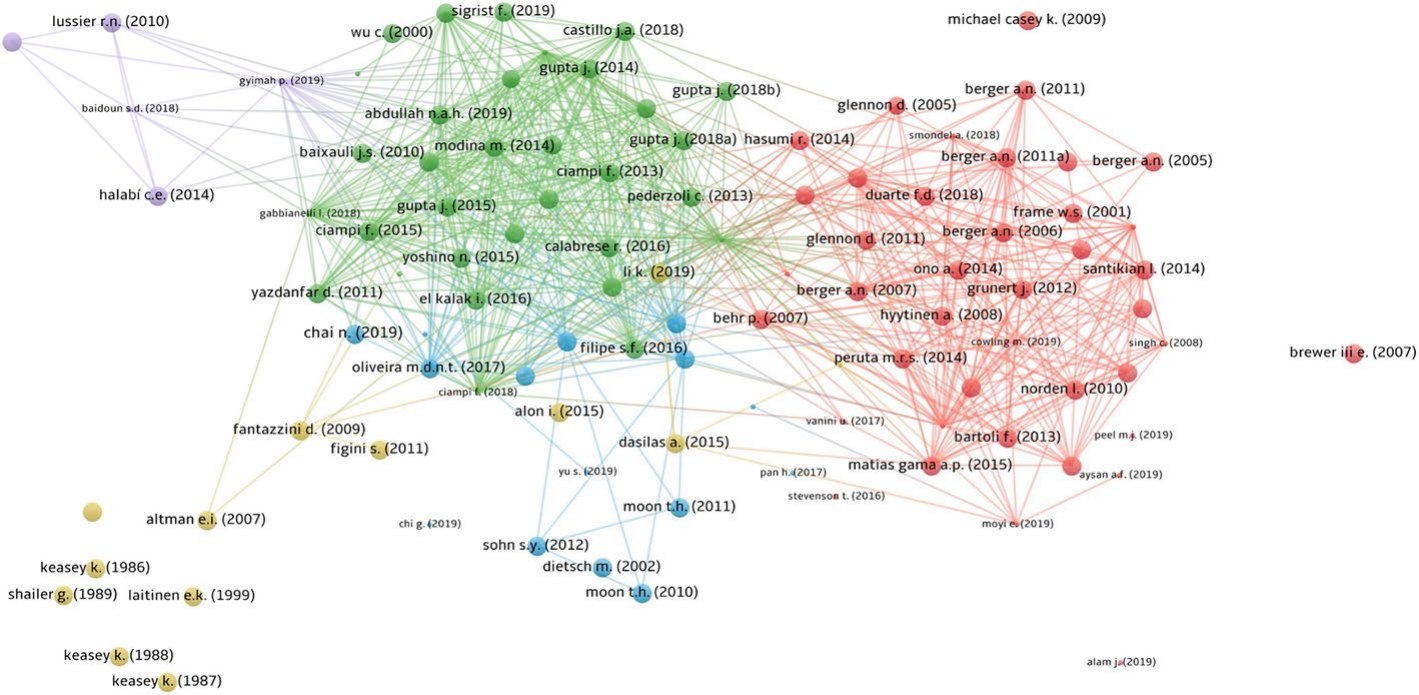
Results of the VOS analysis

Figure 2 contains the graphic output of the VOS analysis. It shows the presence of five well-polarised clusters representing the same number of distinct streams of research within the field of SME default prediction.

Table 2 contains the descriptive statistics concerning each cluster. It shows that Red and Green clusters are the biggest ones in term of total Number of Papers (respectively 41 and 32) and the Red Cluster is the first in terms of Total Citations (962). However, looking at the Total Citations/Number of Papers ratio, the yellow cluster is the most relevant (13 papers collecting 504 citations), followed by the Purple cluster (5 papers collecting 129 citations). Six papers (Altman and Sabato 2007; Angelini et al. 2008; Berger et al. 2005; Berger and Frame 2007; Frame et al. 2001; Keasey and Watson 1987) collect 760 citations, representing the pillars of the entire field of SME default prediction.

**Table 2:** Descriptive statistics concerning the clusters

	Number of papers	Total citations	Total normalised citations	$\frac{{{\text{Total}}\;{\text{citations}}}}{{{\text{Number}}\;{\text{of}}\;{\text{papers}}}}$
Red cluster	41	962	32.2504	23.46
Green cluster	32	218	34.2293	6.81
Blue cluster	15	144	22.0450	9.60
Yellow Cluster	13	504	13.8582	38.77
Purple cluster	5	129	3.8484	25.80

These results confirm that the field of research is rather developed, cited, and relevant. The topics discussed in each cluster can be summarised as follows:

1. The Red cluster focuses on the reciprocal cause-and-effect relationships between default prediction modelling, bank lending activities, and firm-bank relationships, mainly by analysing SME default prediction from the bank perspective.
2. The Green cluster represents the ‘core’ SME default prediction-modelling literature by focusing on the exploration of the prediction potential of numerous quantitative and**,** to a smaller extent, qualitative variables.
3. The Blue cluster explores the potential of innovation-related variables in predicting SME default.
4. The Purple cluster analyses the critical variables for small company success, providing implicit suggestions on the prediction of SME failure.
5. The Yellow cluster focuses on the empirical validation of the seminal theoretical failure-prediction model proposed by Argenti (1976) and the development of SME default-prediction models based on longitudinal data.

Figure 2 also highlights that the two biggest clusters, the Red and the Green ones, are very distinct and not overlapping at all. This result suggests the literature belonging to these clusters is excessively polarised thus showing the need for an integration of the studies adopting a banking approach (Red cluster) with those focused on the exploration of the prediction potential of new qualitative and quantitative variables (Green cluster). One of the goals of the present paper is to facilitate the dialogue and integration between these different and complementary approaches.

**Fig. 3:**
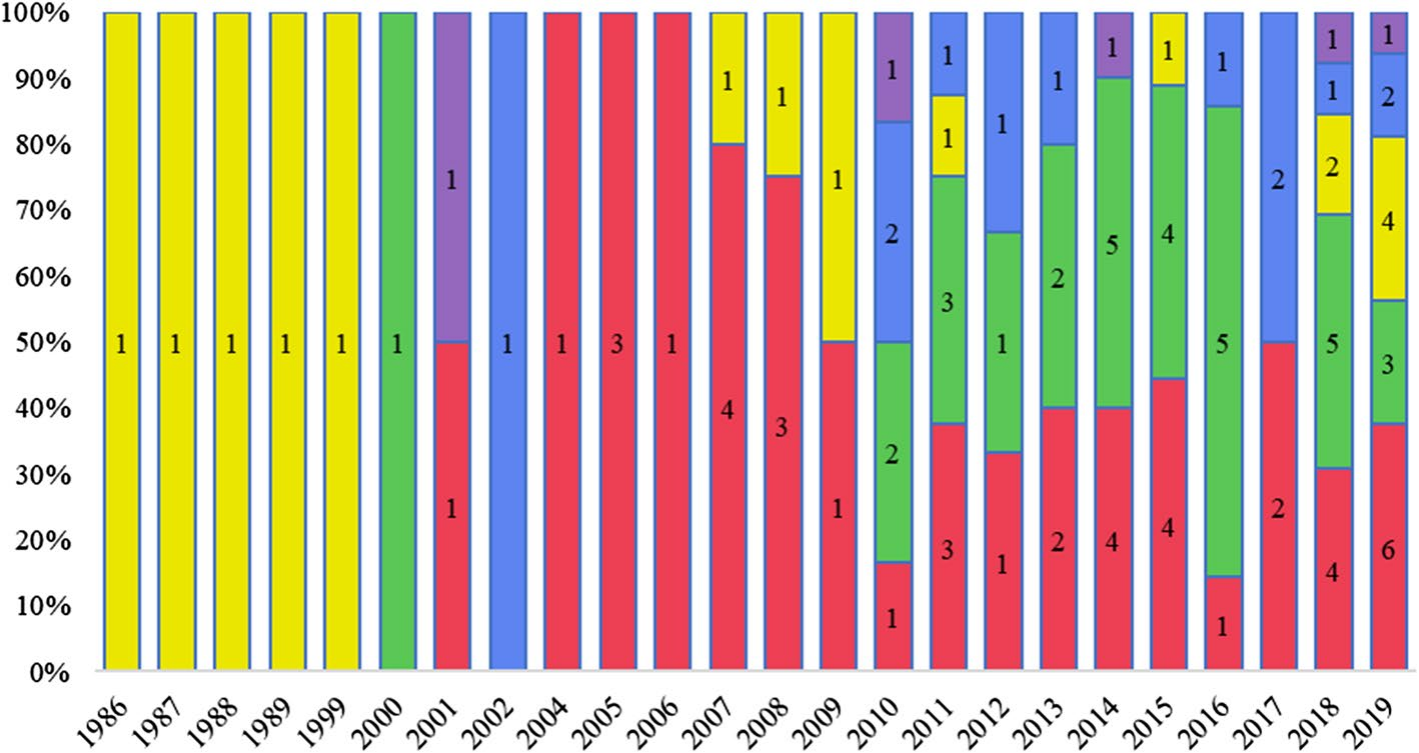
Distribution of papers in the clusters for each year

Figure 3 shows how the papers assigned to each cluster are distributed over time. The colour of the bars represents the colour of the cluster.

The green and the blue clusters have received increased attention and relevance in the last decade. We expect these clusters to grow further in accordance with the research avenues 1 and 3, described below in Sect. 5. Consistent with the analysis in Sect. 4.5, the yellow cluster contains the five least recent studies (subgroup 1 of the cluster) as well as seven of the most recent papers (subgroup 2 of the cluster). The low number of papers in the purple cluster is consistent with the fact that it represents a bridge between the yellow cluster and the most recent studies of the green and blue clusters (see Sect. 4.4, below).

After this preliminary overview, we now move to the detailed review of the papers in each cluster.

### Red cluster: The reciprocal relationships between credit-scoring, bank-lending activities and firm-bank relationships

The traditional types of lending used by banks in their interactions with SMEs are relationship lending, credit score lending and asset-backed lending. Starting from these different lending approaches, the first group of papers consists of articles which do not directly aim at analysing and/or improving the effectiveness of SME default prediction model but are mainly concerned with the reciprocal cause-and-effect relationships between credit-scoring systems and bank-lending activities, with an emphasis on small firm-bank relationships.

Several of these studies (Akhavein et al. 2005; Berger et al. 2005; Berger and Frame 2007; Berger et al. 2011a; Brewer III 2007; DeYoung et al. 2008; Frame et al. 2001; Hasumi and Hirata 2014; Ono et al. 2014; Singh and Griffiths 2008) analyse the relationship between the use of small enterprise (SE) credit-scoring models, SE credit availability and loan pricing. The findings demonstrate that the use of credit scoring determines an increase in the small-business credit availability (Berger et al. 2005; Frame et al. 2001), especially in the case of borrowers who are particularly opaque^2^/risky or located in low‐income areas (Frame and Woosley 2004), greater borrower-bank geographical distances (DeYoung et al. 2008), greater loan maturity (Berger et al. 2005) and a significant reduction of collaterals and in the probability that collateral is pledged (Berger et al. 2011b). Apart from the case of borrower-lender long-distance situations (DeYoung et al. 2008), many studies in this cluster (Behr and Güttler 2007; Berger et al. 2005, 2011b; Peruta et al. 2014) reveal that SEs are more effectively evaluated when banks adopt a relationship lending approach and are consequently better able to acquire the qualitative information which is strategic to assess a SE creditworthiness accurately (Berger 2006).

Berger et al. (2005) highlight that when the credit-scoring lending technology is used to accept or reject credit applications automatically, informational opacity problems are exacerbated, credit terms become less accurate, and lenders face more significant future credit losses. On the contrary, when the relationship between the lender and the borrower is strict, SEs benefit from better credit access, lower interest rates (Chen et al. 2013). In doing this, banks reduce monitoring costs (Hirsch et al. 2018; Sampagnaro et al. 2015) in the SMEs lending, without any significant increase in default rates (Aysan and Disli 2019; Berger et al. 2011a). The positive effect on interest rate generated by strict bank-firm relationship^3^ (longer relationship and a more significant number of accounts) is even more substantial for high-risk SMEs (Neuberger and Räthke-Döppner 2015; Peltoniemi 2007). In addition to the firm-bank relationship variables, the quality of financial reporting and the non-credit services cross-sold to the company exert a significant effect on the cost of credit (DeZoort et al. 2017; Peel 2019; Santikian 2014). Some papers in this cluster (Bartoli et al. 2013; Duarte et al. 2018) also indicate the existence of an unexpected positive relationship between collaterals and bankruptcy for SMEs with high credit-risk levels and the existence of a negative relationship between collateral requirements asked by banks and bank-borrower distance (Bellucci et al. 2019). Other studies (Glennon and Nigro 2005) highlight that the default risk of SMEs is linked to both the regional and industrial economic conditions in which the company operates and to the loan maturity. The existence of a ‘peripheral region price penalty’ in term of lower credit availability and higher cost of credit is confirmed in a recent study based on UK data (Cowling et al. 2019).

Few studies suggest that the traditional SME credit-scoring models based on financial data should be integrated with non-financial variables (Smondel 2018) such as employees' loyalty/satisfaction and long-term relationships with customers (Chen et al. 2013; McCann and McIndoe-Calder 2015) or with credit behavioural information (Norden and Weber 2010; Stevenson and Pond 2016). The use of intellectual capital variables such as management skills, education and expertise of the SME is only suggested in some studies (Grunert and Norden 2012; Vanini and Van Liempd 2017), without including them in default prediction models. The integration of financial information and soft information in quantitative PD models remains an unanswered question in this cluster.

Two recent studies investigate lending activities in micro-finance institutions, suggesting that the probability of default among male borrowers is higher than the default rates of female borrowers (Alam et al. 2019) and that lending to small businesses does not significantly impact on the micro-finance institution risk (Moyi 2019).

### Green cluster: The ‘core’ SME default prediction modelling literature

Among this cluster, the research’s aims are very homogenous and, though using different categories of default predictors and different statistical methods, always consist of the estimation of the best SME default prediction model.

Most of these studies use quantitative variables as default predictors, especially financial ratios, often classified into the three classical categories of leverage, liquidity and profitability (Altman et al. 2020; Baixauli and Módica-Milo 2010; Calabrese et al. 2016; Castillo et al. 2018; Ciampi and Gordini 2013; Gupta et al. 2014a, b; Yazdanfar 2011), as well as macroeconomic variables (Filipe et al. 2016; Kosmidis and Stavropoulos 2014) and company’s age (Abdullah et al. 2016; Lugovskaya 2010). Lin et al. (2012) explore the impact of four different default definitions on the choice of financial predictors and the model's accuracy. They find that profit, growth and employee efficiency are prevalent in all default definitions and that growth in profitability, annual sales and operating revenue are always key variables to predict SME default. Apart from the study of Lin et al. (2012) based on Russian companies, one of the common findings in these contributions is that, among accounting variables, leverage indicators seem to be better predictors of SME default than liquidity and profitability ratios (Modina and Pietrovito 2014). Also, the age and the size of the company are negatively related to the probability of default of SMEs (Abdullah et al. 2019; Altman et al. 2010). Gupta and Gregoriou (2018) find that, although financial variables affecting the bankruptcy probability of both listed and unlisted SMEs are almost identical, listed SMEs have a lower probability of default than unlisted companies, thus suggesting that unlisted SMEs are more vulnerable to changes in financial ratios due to higher capital-rationing constraints.

The vast majority of these studies investigate the most effective financial ratios for SME failure prediction and whether the significant ratios for SMEs are different from those used for larger firms. Another common finding of this group of papers is that prediction models which have been developed for specific industries perform better than generic models (Rikkers and Thibeault 2011) and that using models, specifically estimated on SME samples, increases the default prediction accuracy rates (Altman et al. 2020; Gupta et al. 2015). For example, several authors (El Kalak and Hudson 2016; Gupta et al. 2015, 2018a, b) develop different prediction models for different-sized categories of SMEs (micro, small and medium-sized companies) and find that analysing small and micro firms separately, resulted in an improved default prediction accuracy.

Filipe et al. (2016) proposed an interesting attempt to integrate standard accounting-based models for SME default prediction with the potential of macro-economic variables. They demonstrate that macro variables and the location of the firm affect bankruptcy rates of European SMEs. They also highlight the importance of developing prediction models designed on a regional basis and find that when as SMEs become larger, their vulnerability to macroeconomic factors decreases. They investigate the role of three categories of macroeconomic variables: business cycle related variables, credit conditions (level of interest rates, credit availability) and insolvency codes. They find out that the solidity of the currency, the economic sentiment, and the volumes of bank loans are negatively correlated with the probability of default. In contrast, the unemployment level and the average duration of insolvency proceedings have positively correlated the probability of bankruptcy. Attempting to integrate accounting-based models, Andrikopoulos and Khorasga (2018) propose a ‘hybrid’ default prediction model, combining traditional financial ratios of unlisted SMEs with market information of listed SMEs. Ciampi et al. (2018) highlight the need to design SME prediction models based on quantitative predictors other than financial ratios and macroeconomic variables. Using the Kohonen map-based trajectories, they find that using company prior payment behaviour-related variables improves prediction accuracy, especially for smaller firms and for a horizon longer than one year. Another study (Gabbianelli 2018) suggests that variables related to the characteristics of the territory (degree of attractiveness, articulation of the financial system, orientation to innovation) in which the SMEs operate and the firm–territory relationship (degree of territorial rooting, resources of the territory) exert a significant effect on SME default risk.

A significant number of papers in this cluster (Calabrese et al. 2016; Ciampi et al. 2018; Gupta, Gregoriou, et al. 2018a, b; Yoshino and Taghizadeh-Hesary 2015) also investigate the potential of non-conventional methodologies for default prediction modelling, such as neural networks (Ciampi and Gordini 2013; Mittal et al. 2011; Wu and Wang 2000), Grabit model (Sigrist and Hirnschall 2019) and other operational research methodologies (Calabrese et al. 2016). These studies find that non-parametric models such as neural networks, Kohonen maps, Binary Generalized Extreme Value Additive and Grabit models perform better than traditional logistic regression and Cox proportional hazard, especially at longer horizons. The increase in model prediction accuracy generated by neural networks, in comparison to traditional methodologies, is higher for the smallest-sized firms suggesting that neural networks may enhance small-business lending decisions (Ciampi and Gordini 2013).

Based on the idea that SME default prediction models based only on quantitative variables as default predictors are limited by the opacity of SME financial statements and accounting data (Altman et al. 2010), a limited number of recent studies belonging to this cluster analyse the SME default predictive potential of qualitative predictors (Ciampi 2015; Habachi and Benbachir 2019; Wilson and Altanlar 2014). Wilson and Altanlar (2014) and Ciampi (2015) investigate the effectiveness of using corporate governance characteristics for SME default prediction. Wilson and Altanlar (2014) find that the composition of boards, as well as the background and networks of directors, represent valuable information for small companies default prediction. They also demonstrate that defaulted companies have higher director turnovers, lower numbers of female directors, local directors and family directors, and a lower diffusion of multiple directorships. Ciampi (2015) finds that using corporate governance characteristics (CEO duality, the concentration of ownership, and the number of independent directors) significantly improves SME default prediction accuracy rates.

Another contribution, by Pederzoli et al. (2013),^4^ considers accounting ratios and innovation-based variables for default prediction of SMEs operating in technological industries. Using two innovation-related predictors, this study demonstrates that the value of the patent portfolio reduces the probability of default of innovative SMEs. The dimensions of the patent portfolio are measured by capitalised patent stock divided by capitalised R&D personnel, thereby representing an indicator of the company R&D efficiency. In contrast, the value of the patent portfolio is measured by a multidimensional factor index based on the number of citations received, the number of inventors, the number of offices to which a patent has been presented, and the number of patent classes (International Patent Classifications—IPC). This paper attempts to bridge the gap between innovation literature and default prediction literature. It is precisely this gap that is the specific object of analysis of the papers belonging to the blue cluster.

Summarising the main body of knowledge in this cluster, we may argue that SME default risk depends on traditional financial ratios, age and size of the company, macro-economic factors and characteristics of the territory in which the SME operates, history of payment behaviour (also emerged in the Red cluster), audit qualifications (Altman et al. 2010) and corporate governance information. Even in this cluster, the role of intellectual capital receives little attention, probably due to the problematic integration of financial and qualitative variables in quantitative prediction models, perhaps mainly when scholars use traditional methodologies such as discriminant analysis and logit models as in the majority of papers in this cluster.

### Blue cluster: the potential of innovation-related variables, machine learning/non-linear programming tools and big data for SME default prediction

This group of papers explores the SME default prediction potential of innovation-related variables. It also tests the SME default prediction effectiveness of big data and non-traditional methodologies based on machine learning/non-linear programming.

Several authors (Moon et al. 2011; Moon and Sohn 2010; Sohn et al. 2012; Sohn and Jeon 2010; Sohn and Kim 2013) investigate the relationship between technological innovation and SME default risk. They find that the level of profitable technology owned by a SME together with the legal representatives’ technological knowledge reduces the probability of default, especially by companies in high-tech industries.

One of the most relevant findings in this cluster is to demonstrate the effectiveness of non-financial factors related to product innovation (patents and brand products) as predictive variables together with financial ratios, macroeconomic indicators, and some characteristics of legal representatives such as age, gender and the value of their real estate properties (Chi and Meng 2018; Yu et al. 2019). Few recent studies in this cluster try to solve the methodological issues generated by the need to combine different sources of information to assess SME default risk better, suggesting the use of machine learning/non-linear programming tools such as MCDA (Corazza et al. 2016; Gonçalves et al. 2016), fuzzy clustering (Chai et al. 2019), neural networks (Giannopoulos and Aggelopoulos 2019), non-linear programming with maximum discriminating power of credit scores (Chi et al. 2019) and cognitive mapping (Oliveira et al. 2017).

In combining different sources of information, Gonçalves et al. (2016) develop a multiple criteria decision analysis (MCDA) based system for SMEs credit risk assessment by including, in addition to the traditional financial variables, innovation-related variables (R&D capability and reinvention capability), as well as to human characteristics (honesty, friendliness and ethical posture) of the SME managers, commercial aspects, management experience and skills, and external factors (legislation, financial background and political instability). Oliveira et al. (2017) demonstrate how the SME default predictive power of both financial and qualitative information can be improved by combining MCDA with the use of cognitive mapping. Using non-linear programming tools, Pan (2017) start a promising stream of research by analysing the SME default predictive power of big data related to real transaction-based trade areas. They find that adding a variety of Big Data types, such as those related to credit card sales, improves default prediction accuracy rates, especially for low-risk SMEs.

Despite the consistent efforts of recent studies in this cluster to use innovation-related variables and, in general, to combine different sources of information (such as Big Data) in credit rating models with the use of machine learning tools, these models are tested only at local levels and a generalised and validated quantitative model is still missing.

### Purple cluster: SME success versus failure prediction models

This cluster concerns the identification of critical variables for small business (SE) success using survey analysis as the primary research methodology. This research stream aims to achieve a better understanding of those resources which are relevant to small firm development and success, thus providing indirect suggestions on the prediction of small firm failure. The main contribution of this cluster is to highlight the crucial role of human capital in business success.

Lussier and Pfeifer (2001) investigate the role of 15 SE organisational variables and owners’ characteristics and demonstrate that US SEs significantly increase their probability of success when they blend adequate capitalisation, control and planning systems, together with an adequate education level of the owner and experienced and skilled entrepreneurs. The validity of the 15-variables model developed by Lussier and Pfeifer for the US SMEs has been confirmed in predicting the business success of firms located in other countries such as Chile, Croatia, Palestine and Ghana (Baidoun et al. 2018; Gyimah et al. 2019; Lussier and Halabi 2010), taking a partial step toward the creation of an empirically validated theoretical framework for a cross-national SE success prediction model based on non-financial information.

Halabì and Lussier (2014) extend the Lussier findings by demonstrating that having adequate levels of working capital, financial control and planning processes, making use of adequately skilled advisors, together with an adequate education level of the owner, significantly increases a SE probability of success.

As it proposes SME success prediction models mainly based on qualitative variables, this cluster paves the way for overcoming the classical credit-scoring lending models, traditionally based on accounting figures and ratios, and represents a bridge between subgroup 1 of the yellow cluster and the most recent studies of the green and blue clusters which propose the integration of qualitative, non-accounting-based variables into the SME default prediction modelling literature.

Nevertheless, concerning critical variables for SME success/failure, there is still relevant discordance in the literature between theoretical studies as well as between empirical findings from different countries. Even though the Lussier model has been tested in five different countries (US, Chile and Croatia, Ghana and Palestine), its ability to predict small business success/failure remains relatively low.

### Yellow cluster: the empirical validation of Argenti’s theoretical model and SME default prediction models based on longitudinal data

We divided this cluster into two subgroups.

#### Subgroup 1: the empirical validation of Argenti’s theoretical model

The studies belonging to this subgroup focus on the empirical validation of the seminal theoretical failure prediction model proposed by Argenti (1976).

Several of these studies (Keasey and Watson 1986, 1987, 1988; Laitinen and Gin Chong 1999) propose a SME bankruptcy prediction model based on financial predictors as well as on many non-financial variables extracted from Argenti’s model. They find that using variables related to the number of directors (Dasilas and Papasyriopoulos 2015), directorship changes, audit qualifications, the effectiveness of the financial control/planning system, parent firm’s characteristics in franchising chains (Alon et al. 2015) and the presence of loans secured by the firm’s assets makes it possible to achieve better prediction rates than using financial ratios only. The positive relationship between the presence of loans secured by the firm’s assets and SME’s performance/survival is confirmed in a recent study by (Gharsalli 2019).

One of the main contributions of this group of papers is the identification of critical early-warning signals of SMEs failure such as the ‘incompetence of management’, followed by ‘deficiencies in accounting systems’ (thus demonstrating the importance of internal financial control and planning systems) and ‘attitude towards customers’ (Laitinen and Gin Chong 1999).

#### Subgroup 2: developing SME default prediction model using longitudinal data

The papers in this subgroup propose SME default prediction models based on longitudinal data.

From a methodological perspective, Angelini et al. (2008) confirm the usefulness of non-linear methodologies for SME default prediction by developing a SME rating model based on neural networks fed by a combination of financial and credit behavioural longitudinal data, while Fantazzini and Figini (2009) demonstrate that Bayesian longitudinal models increase the in-sample prediction accuracy in comparison to traditional pooled logit models.

Figini and Giudici (2011) develop a methodology to merge longitudinal models. They use both financial variables (such as the capability of a SME to receive non-bank financing, the degree of financial leverage and the return on assets ratio) and qualitative predictors (such as the payment history, the company life cycle phase and the situation regarding orders from customers^5^). They find that the merged longitudinal model (which combines the scores separately obtained from financial and qualitative variable to arrive at the definitive probability of default of each SME) performs better than models based on only one category of independent variables.

Incorporating behavioural/qualitative variables in quantitative models based on traditional methodologies (discriminant analysis and logit regression) is challenging (Altman and Sabato 2007; Shailer 1989). Starting from this limitation of traditional quantitative techniques, the main contribution of this group of papers is the application of non-traditional methodologies (neural networks and Bayesian longitudinal models) to a longitudinal dataset and to merge different categories of predictive variables. This represents the ‘quantitative’ contribution that statisticians provide to improve SMEs prediction models, trying to solve one of the most relevant methodological issues in default prediction: the combination of scores estimated from models based on different categories of information. Despite the relevant methodological contribution, they use only a few of the variables proposed in the Subgroup 1 of this cluster (i.e. the attitude towards customers), and they do not consider the role of human capital and the innovation-related variables as proposed in the Purple and Blue cluster, respectively.

## SME default prediction: a research agenda

### Research avenue 1: enlarging the set of qualitative variables for SME default prediction

Although several studies included in the green, blue and purple clusters and in subgroup 1 of the yellow cluster have already directly (blue and green clusters) or indirectly (purple and yellow clusters) theorised and tested the predictive power of non-financial variables for SME bankruptcy prediction, the permanence of significant errors highlights the need to increase the categories of SME default predictors, intending to include the highest possible number of qualitative variables. Two main areas of interest should be considered in this connection, the internal characteristic of the SME and the network ties where the SME is embedded.

Regarding the first area, the literature identified several reliable predictors of SMEs performance such as the management capabilities in developing an ambidextrous approach to competition (Lubatkin et al. 2006), the characteristics of the intellectual capital possessed by the firm (Cohen and Kaimenakis 2007), the organisational learning ability in making the knowledge possessed by the firm at work (Hsu and Fang 2009), and the innovation ability of the firm (Rosenbusch et al. 2011). Regarding SMEs performance predictors related to the network ties where the SME is embedded the literature identified variables such as the number international strategic alliances (Lu and Beamish 2001), and the features of cooperation networks (Zeng et al. 2010) and home-based social networks (Zhou et al. 2007).

However, the pivotal issue for the present research avenue is related to how one can easily measure the aforementioned variables (or proxies) to infer such SMEs characteristics in a fast, efficient and cost-effective way.

SMEs have lighter obligations concerning accounting data disclosure and they also have automatically smaller accounting figures when compared to more prominent companies. As a result, values and trends of some financial ratios become not at all significant below certain size thresholds (Ciampi 2015). Furthermore, in the case of SMEs, the impact of external events (e.g. loss of a significant client or a key manager or a wrong strategic decision) is higher, and it may suddenly weaken a state of financial soundness by harming a firm’s strengths (Ciampi^2018^). These reasons render quantitative variables in general and financial ratios in particular extremely weak as SME default predictors (Ciampi and Gordini 2013). Consequently, even if the effort required by scholars and practitioners to address such issues seems largely demanding, the present research avenue appears very promising.

### Research avenue 2: global SME default prediction modelling

Although some studies included in the green cluster tested the use of macroeconomic information for SE default prediction, most scholars have so far assumed that national models perform better than international ones, since they are better able to capture the peculiarities of SMEs located in a specific national context. As a matter of fact, in the literature, significant differences between countries have been found in the critical variables for success/failure of SMEs (Lussier and Halabi 2010). The specific features, in terms of regulations, financial and industrial structure, of a single national economic system (capital market orientation, the role played by banks; efficiency of the stock exchange markets, degree of ownership concentration) have a considerable impact upon which are the most significant SME default predictors and also on the ways through which these variables affect.

Further studies should, therefore, include cross-country analysis, in order to see if and how the predictive capacity of the different variables in a particular national context can also be effectively exploited in ultra-national economic and industrial contexts. On the other hand, a deeper understanding of the heterogeneity of the vital predictive variables in the different countries would be fundamental to develop an ultra-national SME default prediction model. Considering the increased globalisation and interconnection between economies and companies located in different countries, we encourage scholars to develop a globally-tested and validated SME default prediction model by including cultural and environmental variables, regional macroeconomic variables (such as the evolution of the local currency, growth of GDP, unemployment trends and exchange rate trends) and regional credit conditions (such as level of interest rates and credit availability), in order to control for the differences among different countries.

*Is it possible to build an ultra-national SME default prediction model that applies globally? How can we control for cultural differences, credit conditions and macroeconomic variables in different countries?*

### Research avenue 3: innovation based SME default prediction modelling

In an environment with increasing competition, the innovation skills are more and more fundamental for the SMEs’ success and failure, as suggested by some papers included in the blue cluster (Moon et al. 2011; Moon and Sohn 2010; Sohn et al. 2012; Sohn and Jeon 2010; Sohn and Kim 2013), which have investigated the predictive power of a limited number of innovation-related variables.

Nevertheless, it is still unclear which variables to use, how to measure these variables accurately, and how to effectively integrate them in an adequately articulated SME default prediction model. Pederzoli et al. (2013) attempted to demonstrate the contribution of the innovation-related variables to SME default prediction by using patents data from 17 European countries. The authors themselves, however, stressed that patents are only a limited measure as SMEs usually hold a minimal number of patents. Moreover, as emphasised by Andries and Fames (2013), the number of patents is generally associated with a positive financial performance of SMEs only in the long-run.

On the side of innovation literature, scholars have traditionally debated around how and to what extent innovation is associated with a positive performance of SMEs. In doing so, this literature provided numerous measurable variables significantly related to SMEs innovation performance.

A recent meta-analysis by Rosenbusch et al. (2011) found that the mere use of variables such as the number of new products or patents is not particularly significant to measure the SME innovation performance properly. Contextual factors such as the category of innovation, the cultural context, the age of the firm, and network ties affect SMEs innovation performance much more than the number of new products or patents.

Literature about innovation also highlighted another difference in measuring innovation capabilities between SMEs and large companies. While the amount of expenditure devoted to R&D activities is a reliable and easy-to-measure variable in the case of large companies, it does not seem to be a robust measure in the case of SMEs (Wolff 2007). On this side, diligence and the use of appropriate internal management tools (such as ISO quality certifications) suitable for adequately formalising structured innovation development process seem to be more effective antecedents of SMEs innovation performance (Howell et al. 2005). Rosenbusch et al. (2011) also stressed that SMEs innovation performance is significantly associated with the strategic innovation orientation of the company that ultimately results in higher brand reputation, more effective collaboration from partners, and attracting highly-competent employees.

Moving from these research findings in the SME innovation literature, the main goal for scholars and practitioner in the present area of study is to identify a series of variables (such as the innovation skills of the SMEs management, the SMEs internal R&D characteristics, and the SMEs collaborative innovation abilities) related to the SMEs innovation capability, measure these variables in a cost-effective way, and use them for SME default prediction.

As most of the innovation performance variables proposed in the abovementioned innovation literature, such as strategic orientation, entrepreneurial variables, internal development process, and network ties, are difficult to gather from companies’ financial statements and require high investments to be collected, as, in the case of the research avenue 1, the biggest challenge for the present line of research is to measure efficiently and effectively the aforementioned variables.

In facing this challenge, the use of automatic tools such as Big Data and Neural Networks (reviewed in the green and yellow clusters) will play a crucial role in helping to acquire soft information with a cost-efficient and prediction effective approach. Therefore, the present research avenue goes hand by hand with the next one (research avenue 4), and we believe that they will grow exponentially in the next years by benefiting from the contribution of areas of research such as Data Science, Computer Science, and Information Systems.

### Research avenue 4: exploring the potential of Big Data for SME default prediction modelling

Consistent with the kind of data that financial institutions can provide without privacy concerns, most of the existing research on SME default prediction is based on substantially small samples composed by homogenous firms and traditional data-processing techniques (Chen 2009, 2012). This has two crucial consequences on researchers’ outputs. First, most of the models are not fine-tuned due to the restrictions deriving from existing statistical instruments (Tseng and Hu 2010). Second, the fact that computation times are often incredibly long represents a relevant limitation of these studies in term of practical implications. As an example, Altman, Marco and Varetto (Altman et al. 1994) based their research on 1000 firms using 1000 Artificial Neural Networks’ learning cycles to achieve a suitable model. Nowadays, these issues could be reduced by using big data analytics (BDA). Big data are datasets that are too complex to be managed through traditional data-processing software (Fosso Wamba et al. 2015). BDA are an ensemble of tools and procedures capable of treating and analysing such datasets (Fosso Wamba et al. 2015). Also, the use of BDA involves the utilisation of machine learning, supported vector machines (SVM), random forests, Bayesian techniques, and artificial intelligence (AI).

Compared to traditional data-processing techniques, BDA are capable of reducing both computational times (thanks to cloud computing, machine learning and AI) and researchers’ biases regarding which parameters have to be considered to improve models (Fosso Wamba et al. 2015). Indeed, several finance and marketing researches have demonstrated that BDA can be effectively used to develop accurate predictive models that can represent excellent support for financial decision making (Bukovina 2016) and default prediction modelling (Alaka et al. 2018).

Considering that accuracy rates of traditional default prediction models are meagre when applied to small and medium-sized firms (Ciampi 2015), this potential could also be interestingly exploited to develop default prediction models specific to SMEs, allowing researchers and practitioners to include new kinds of data that are non-manageable with traditional techniques (i.e. credit card transactions or commercial transactions in the district in which the SE is located). Also, we strongly suggest scholars to include unstructured big data inputs, transform them into structured variables and feed them into prediction models.

The investigation of the role of positive and negative press coverage and social media data could become a priority in the SMEs credit risk assessment. Traditional default prediction models could be enhanced by (1) introducing artificial intelligence techniques to provide real-time ‘soft’ variables such as governance and social media derived measures and (2) by additional components capturing macro-economic data from the country and industrial sector of the SME. This data enabled the models to adapt to relevant environmental-economic conditions, like GDP growth, unemployment, and sector specific default rates, if available.^6^

As already demonstrated by one of the studies belonging to the blue cluster (Pan et al. 2017), these methodologies could allow the effective treatment of unstructured datasets and the identification of hidden patterns concerning SME default signals. As an example, BDA techniques based on AI and machine learning, allowed to find out that unexpected and usually neglected parameters matter for default prediction (Alaka et al. 2018; Pan et al. 2017).

As SMEs frequently have a minimal digital footprint (i.e. no social media pages, rudimental websites, and limited financial information available online), the biggest challenge for the present line of research is related to how to dispose of enough collectable Big Data. Financial institutions, on the other hands, possess extensive datasets about their customers that could be effectively analysed through BDA, machine learning, and AI. This research avenue is almost unexplored, but we expect it will become one of the main topics in the field due to the extremely fast-growing pace of the application of BDA, machine learning and AI to the business and financial fields (Jordan and Mitchell 2015).

### Research avenue 5: including credit-relationship variables in SME default prediction models

Several studies belonging to the red cluster (Chen et al. 2013; Neuberger and Räthke-Döppner 2015) demonstrated a strict relationship between the lender and the borrower enhances the SE ability to access to credit as well as to bargain low interest rates and fewer collateral requirements. This is due to the soft information gained by lenders thanks to the strict, direct and continuous relationships developed with the client firm. This notwithstanding, the effect of the inclusion of lender-borrower relationship-related variables in SME default prediction modelling is still largely unexplored. Thus, we encourage scholars to select and test all the default prediction variables which are engrained in the relationship lending context (such as duration of the relationship, the processing time of loans, the number of firm’s debit accounts, the number of firm’s active accounts and the number of firm’s loans) or, more in general, which are connected to the reduction of the information asymmetries between borrower and lender (which variables impact on how the lenders ‘understand’ the business of the borrowers). This analysis could also be done using unstructured data from expert opinions.

Examples of unanswered questions are: *How can we measure the degree of information asymmetry between lenders and borrowers? How can we measure the substance, characteristics and effectiveness of the SME-bank relationship? Which is the SME default prediction potential of relationship-lending variables?*

### Research avenue 6: merging different categories of SME default prediction variables

Assuming the lender perspective, it would be precious to test and use all the different category of predictive variables proposed by the studies included in all the clusters identified in this study and then combine the models based on each category of variables in order to develop a multiple default prediction model. A study included in subgroup 2 of the yellow cluster (Figini and Giudici 2011) developed a methodology for data-merging in longitudinal models; but it used only a few variables (financial ratios and payment history). No study has instead investigated the combination of multiple scores coming from a consistent number of categories of predictive information. Thus, we encourage scholars to explore and measure the predictive power of different variables and/or category of variables: from financial ratios to firm payment behaviour-related variables; from corporate governance variables to management and organisational skills-related variables; from innovation-related variables to bank-firm relationship variables; and then to merge the predictive power of each variable and/or category of variables (through appropriate weights and scores), using, for example, the Bayesian methodology, as proposed by Figini and Giudici (2011). In this suggested avenue, it also appears to be valuable, the use of a dynamic event-history analysis by the use of non-traditional methodologies (machine learning/non-linear programming tools) that could allow investigating how the time dynamics impact on the relationship between SME default predictive variables and SME bankruptcy (Ciampi 2015).

As we expect that a multiple default prediction system based on a different category of predictive information would perform better than a system based on a single or a few categories of variables, the present research avenue might be the richest one in term of managerial implications. This research avenue appears extremely valuable in the post-COVID-19 economic environment in which higher level of uncertainty will make financial ratios even less useful than in the past to assess SMEs creditworthiness and the probability to survive will mainly depend on qualitative information, innovation skills and human capital.

## Conclusion

This study constitutes the first attempt to carry out a comprehensive, systematic and detailed review of the literature on SME default prediction. As a result of the bibliometric analysis, we identify five well-polarised clusters and find that the bulk of the research conducted to date on SME default prediction has developed around some defined research themes: the reciprocal cause-and-effect relationships between default prediction modelling, bank lending activities, and firm-bank relationships (analysed mainly from the bank perspective); the estimation of the best possible SME default prediction model by using different statistical methods and exploring the prediction potential of a large number of quantitative and, more recently, also qualitative variables (the ‘core’ SME default prediction modelling literature); the specific exploration of the prediction potential of innovation-related variables; the identification of the critical variables for a SME success, which represent fundamental conceptual antecedents of a SME default predictors; the empirical validation of the seminal theoretical failure prediction model proposed by Argenti (1976); and the development of SME default prediction models mainly based on longitudinal data.

Consistent with the abovementioned themes and the connections between papers and clusters, our study shows that future research on SME default prediction can address several promising issues: enlarging the set of qualitative predictive variables by including a more significant number of SME internal characteristic (such as management’s ambidextrous capabilities and the characteristics of the intellectual capital possessed by the firm), as well the characteristics of the network ties where the SME is embedded (such as the number international strategic alliances of the firm); developing a globally (ultra-national) validated SME default prediction model; deepening the analysis of the prediction potential of a series of innovation-related variables and effectively integrating these variables in the SME default prediction models; exploring the SME default prediction potential of big data (unstructured data such as negative or positive press coverage or social media) and big data analytics; exploring the prediction potential of credit-relationship based variables (such as the duration of the lender-borrower relationship, the processing time of loans or the number of firm’s debt accounts); combining the multiple scores coming from a consistent number of categories of predictive information.

These results suggest the existence of many opportunities to improve the knowledge on SME default prediction, paving the way to direct the changes of rating inputs imposed by the Covid-19 crisis towards more extensive use of qualitative variables/soft information, related to intellectual/human capital and innovation skills. As they are expected to be extremely promising for improving credit-risk management decision-making by banks and other financial intermediaries in the new economic scenario, the implications of these opportunities appear relevant not only from an academic standpoint but also from the practitioner perspective.

The main limitation of the present study is linked to the nature of the bibliometric analysis that naturally tends to simplify the structure and characteristics of a field of research. We tried to compensate this limitation by proposing a systematic literature review of the papers belonging to each cluster, which allowed us to grasp the content and methodological connections between papers and research streams. Indeed, the present paper, reviewing 106 documents, cannot encompass the complexity of the findings presented in each paper; instead, it should be considered as a guide in the tangled forest of the research around SMEs’ default prediction and a starting point for future research on the topic.

## Appendix

In the present appendix we included all the papers considered in the present study. The papers are ordered by Cluster size on a first instance, Normalised Citations on the second instance, and Total Citations on the third instance. Please note that Angilella and Mazzù (2019), Cornée (2019), Edmister (1972), Laitinen (1993), Liu et al. (2019) were not connected in term of shared references and consequently excluded from the similarity analysis.

**Table:**

VOS label	Authors	Title	Journal	Year	Cluster	Normalized citation	Total citations	Link to full text
berger a.n. (2011a)	Berger a.n.; espinosa-vega m.a.; frame w.s.; miller n.h	Why do borrowers pledge collateral? New empirical evidence on the role of asymmetric information	Journal of financial intermediation, 20(1), 55–70	2011	Red	3.7647	56	https://doi.org/10.1016/j.jfi.2010.01.001
norden l. (2010)	norden l.; weber m	Credit line usage, checking account activity, and default risk of bank borrowers	Review of financial studies, 23(10), 3665–3699	2010	Red	2.2381	47	https://doi.org/10.1093/rfs/hhq061
duarte f.d. (2018)	duarte f.d.; gama a.p.m.; gulamhussen m.a	Defaults in bank loans to smes during the financial crisis	Small business economics, 51(3), 591–608	2018	Red	2.1667	1	https://doi.org/10.1007/s11187-017-9944-9
santikian l. (2014)	santikian l	The ties that bind: bank relationships and small business lending	Journal of financial intermediation, 23(2), 177–213	2014	Red	2.0968	13	https://doi.org/10.1016/j.jfi.2013.11.004
berger a.n. (2011)	berger a.n.; cowan a.m.; frame w.s	The surprising use of credit scoring in small business lending by community banks and the attendant effects on credit availability, risk, and profitability	Journal of financial services research, 39(1–2), 1–17	2011	Red	2.0168	30	https://doi.org/10.1007/s10693-010-0088-1
berger a.n. (2005)	berger a.n.; frame w.s.; miller n.h	Credit scoring and the availability, price, and risk of small business credit	Journal of money, credit and banking, 37(2), 191–222	2005	Red	1.9200	128	https://doi.org/10.1353/mcb.2005.0019
deyoung r. (2008)	deyoung r.; glennon d.; nigro p	Borrower-lender distance, credit scoring, and loan performance: evidence from informational-opaque small business borrowers	Journal of financial intermediation, 17(1), 113–143	2008	Red	1.5966	93	https://doi.org/10.1016/j.jfi.2007.07.002
berger a.n. (2007)	berger a.n.; frame w.s	Small business credit scoring and credit availability	Journal of small business management, 45(1), 5–22	2007	Red	1.4731	104	https://doi.org/10.1111/j.1540-627x.2007.00195.x
peruta m.r.s. (2014)	peruta m.r.s.; campanella f.; giudice m.d	Knowledge sharing and exchange of information within bank and firm networks: the role of the intangibles on the access to credit	Journal of knowledge management, 18(5), 1036–1051	2014	Red	1.4516	9	https://doi.org/10.1108/jkm-06-2014-0255
grunert j. (2012)	grunert j.; norden l	Bargaining power and information in sme lending	Small business economics, 39(2), 401–417	2012	Red	1.4423	25	https://doi.org/10.1007/s11187-010-9311-6
frame w.s. (2001)	frame w.s.; srinivasan a.; woosley l	The effect of credit scoring on small-business lending	Journal of money, credit and banking, 33(3), 813–825	2001	Red	1.2038	127	https://doi.org/10.2307/2673896
neuberger d. (2015)	neuberger d.; räthke-döppner s	The role of demographics in small business loan pricing	Small business economics, 44(2), 411–424	2015	Red	1.1928	11	https://doi.org/10.1007/s11187-014-9602-4
frame w.s. (2004)	frame w.s.; woosley l	Credit scoring and the availability of small business credit in low-and moderate-income areas	Financial review, 39(1), 35–54	2004	Red	1.0000	44	https://doi.org/10.1111/j.0732-8516.2004.00066.x
berger a.n. (2006)	berger a.n	Potential competitive effects of basel ii on banks in sme credit markets in the united states	Journal of financial services research, 29(1), 5–36	2006	Red	1.0000	34	https://doi.org/10.1007/s10693-005-5106-3
akhavein j. (2005)	akhavein j.; frame w.s.; white l.j	The diffusion of financial innovations: an examination of the adoption of small business credit scoring by large banking organizations	Journal of business, 78(2), 577–596	2005	Red	0.9450	63	https://doi.org/10.1086/427639
bartoli f. (2013)	bartoli f.; ferri g.; murro p.; rotondi z	Bank-firm relations and the role of mutual guarantee institutions at the peak of the crisis	Journal of financial stability, 9(1), 90–104	2013	Red	0.8861	14	https://doi.org/10.1016/j.jfs.2012.03.003
matias gama a.p. (2015)	matias gama a.p.; van auken h	The interdependence between trade credit and bank lending: commitment in intermediary firm relationships	Journal of small business management, 53(4), 886–904	2015	Red	0.8675	8	https://doi.org/10.1111/jsbm.12115
ono a. (2014)	ono a.; hasumi r.; hirata h	Differentiated use of small business credit scoring by relationship lenders and transactional lenders: evidence from firm-bank matched data in japan	Journal of banking and finance, 42(1), 371–380	2014	Red	0.8065	5	https://doi.org/10.1016/j.jbankfin.2014.02.009
behr p. (2007)	behr p.; güttler a	Credit risk assessment and relationship lending: an empirical analysis of german small and medium-sized enterprises	Journal of small business management, 45(2), 194–213	2007	Red	0.6799	48	https://doi.org/10.1111/j.1540-627x.2007.00209.x
hyytinen a. (2008)	hyytinen a.; pajarinen m	Opacity of young businesses: evidence from rating disagreements	Journal of banking and finance, 32(7), 1234–1241	2008	Red	0.5665	33	https://doi.org/10.1016/j.jbankfin.2007.10.006
michael casey k. (2009)	michael casey k.; selwyn ellis t.s.; linn g.; griffin k	Post-loan credit risk: an analysis of small business in southern arkansas	Competitiveness review, 19(4), 342–348	2009	Red	0.5000	1	https://doi.org/10.1108/10595420910977443
hasumi r. (2014)	hasumi r.; hirata h	Small business credit scoring and its pitfalls: evidence from japan	Journal of small business management, 52(3), 555–568	2014	Red	0.4839	3	https://doi.org/10.1111/jsbm.12049
peltoniemi j. (2007)	peltoniemi j	The benefits of relationship banking: evidence from small business financing in finland	Journal of financial services research, 31(2–3), 153–171	2007	Red	0.3399	24	https://doi.org/10.1007/s10693-007-0009-0
sampagnaro g. (2015)	sampagnaro g.; meles a.; verdoliva v	Monitoring in small business lending: how to observe the unobservable	Journal of financial research, 38(4), 495–510	2015	Red	0.3253	3	https://doi.org/10.1111/jfir.12082
mccann f. (2015)	mccann f.; mcindoe-calder t	Firm size, credit scoring accuracy and banks’ production of soft information	Applied economics, 47(33), 3594–3611	2015	Red	0.3253	3	https://doi.org/10.1080/00036846.2015.1019034
chen y. (2013)	chen y.; huang r.j.; tsai j.; tzeng l.y	Soft information and small business lending	Journal of financial services research, 47(1), 115–133	2013	Red	0.3165	5	https://doi.org/10.1007/s10693-013-0187-x
glennon d. (2011)	glennon d.; nigro p	Evaluating the performance of static versus dynamic models of credit default: evidence from long-term small business administration-guaranteed loans	Journal of credit risk, 7(2), 3–35	2011	Red	0.2689	4	https://doi.org/10.21314/jcr.2011.125
brewer iii e. (2007)	brewer iii e	On lending to small firms	Journal of small business management, 45(1), 42–46	2007	Red	0.2408	17	https://doi.org/10.1111/j.1540-627x.2007.00197.x
glennon d. (2005)	glennon d.; nigro p	An analysis of sba loan defaults by maturity structure	Journal of financial services research, 28(1–3), 77–111	2005	Red	0.1350	9	https://doi.org/10.1007/s10693-005-4357-3
moyi e. (2019)	moyi e	Riskiness of lending to small businesses: a dynamic panel data analysis:	Journal of risk finance	2019	Red	0.0000	0	https://doi.org/10.1108/jrf-09-2017-0140
aysan a.f. (2019)	aysan a.f.; disli m	Small business lending and credit risk: granger causality evidence	Economic modelling, 83, 245–255	2019	Red	0.0000	0	https://doi.org/10.1016/j.econmod.2019.02.014
bellucci a. (2019)	bellucci a.; borisov a.; giombini g.; zazzaro a	Collateralization and distance	Journal of banking and finance, 100, 205–217	2019	Red	0.0000	0	https://doi.org/10.1016/j.jbankfin.2019.01.011
cowling m. (2019)	cowling m.; lee n.; ughetto e	The price of a disadvantaged location: regional variation in the price and supply of short-term credit to smes in the uk	Journal of small business management	2019	Red	0.0000	0	https://doi.org/10.1080/00472778.2019.1681195
alam j. (2019)	alam j.; moir r.; ibn boamah m	Gender and micro-credit: who repays? Evidence from a canadian individual-lending approach	Journal of small business and entrepreneurship	2019	Red	0.0000	0	https://doi.org/10.1080/08276331.2019.1606966
peel m.j. (2019)	peel m.j	The impact of filing micro-entity accounts and the disclosure of reporting accountants on credit scores: an exploratory study	Accounting and business research, 49(6), 648–681	2019	Red	0.0000	0	https://doi.org/10.1080/00014788.2018.1493374
hirsch b. (2018)	hirsch b.; nitzl c.; schoen m	Interorganizational trust and agency costs in credit relationships between savings banks and smes	Journal of banking and finance, 97, 37–50	2018	Red	0.0000	0	https://doi.org/10.1016/j.jbankfin.2018.09.017
smondel a. (2018)	smondel a	Smes' soft information and credit rationing in france	Human systems management, 37(2), 169–180	2018	Red	0.0000	0	https://doi.org/10.3233/hsm-17180
dezoort f.t. (2017)	dezoort f.t.; wilkins a.; justice s.e	The effect of sme reporting framework and credit risk on lenders' judgments and decisions	Journal of accounting and public policy, 36(4), 302–315	2017	Red	0.0000	0	https://doi.org/10.1016/j.jaccpubpol.2017.05.003
vanini u. (2017)	vanini u.; van liempd d	Intellectual capital and banks' credit assessment of smes: evidence from denmark and germany	International journal of learning and intellectual capital, 14(3), 252–276	2017	Red	0.0000	0	https://doi.org/10.1504/ijlic.2017.086393
stevenson t. (2016)	stevenson t.; pond k	Sme lending decisions – the case of uk and german banks: an international comparison	Studies in economics and finance, 33(4), 501–508	2016	Red	0.0000	0	https://doi.org/10.1108/sef-12-2014-0243
singh c. (2008)	singh c.; griffiths m.d	The role of computer usage in the availability of credit for small businesses	Managerial finance, 34(2), 103–115	2008	Red	0.0000	0	https://doi.org/10.1108/03074350810841295
gupta j. (2018a)	gupta j.; gregoriou a.; ebrahimi t	Empirical comparison of hazard models in predicting smes failure	Quantitative finance, 18(3), 437–466	2018	Green	6.5000	3	https://doi.org/10.1080/14697688.2017.1307514
abdullah n.a.h. (2019)	abdullah n.a.h.; ahmad a.h.; zainudin n.; rus r.m	Predicting financially distressed small- and medium-sized enterprises in malaysia	Global business review, 20(3), 627–639	2019	Green	2.4286	2	https://doi.org/10.1177/0972150919837053
ciampi f. (2013)	ciampi f.; gordini n	Small enterprise default prediction modeling through artificial neural networks: an empirical analysis of italian small enterprises	Journal of small business management, 51(1), 23–45	2013	Green	2.3418	37	https://doi.org/10.1111/j.1540-627x.2012.00376.x
ciampi f. (2015)	ciampi f	Corporate governance characteristics and default prediction modeling for small enterprises. An empirical analysis of italian firms	Journal of business research, 68(5), 1012–1025	2015	Green	2.1687	20	https://doi.org/10.1016/j.jbusres.2014.10.003
castillo j.a. (2018)	castillo j.a.; mora-valencia a.; perote j	Moral hazard and default risk of smes with collateralized loans	Finance research letters, 26, 95–99	2018	Green	2.1667	1	https://doi.org/10.1016/j.frl.2017.12.010
gupta j. (2018b)	gupta j.; gregoriou a	Impact of market-based finance on smes failure	Economic modelling, 69, 13–25	2018	Green	2.1667	1	https://doi.org/10.1016/j.econmod.2017.09.004
el kalak i. (2016)	el kalak i.; hudson r	The effect of size on the failure probabilities of smes: an empirical study on the us market using discrete hazard model	International review of financial analysis, 43, 135–145	2016	Green	1.7500	12	https://doi.org/10.1016/j.irfa.2015.11.009
gupta j. (2015)	gupta j.; gregoriou a.; healy j	Forecasting bankruptcy for smes using hazard function: to what extent does size matter?	Review of quantitative finance and accounting, 45(4), 845–869	2015	Green	1.6265	15	https://doi.org/10.1007/s11156-014-0458-0
calabrese r. (2016)	calabrese r.; marra g.; osmetti s.a	Bankruptcy prediction of small and medium enterprises using a flexible binary generalized extreme value model	Journal of the operational research society, 67(4), 604–615	2016	Green	1.4583	10	https://doi.org/10.1057/jors.2015.64
filipe s.f. (2016)	filipe s.f.; grammatikos t.; michala d	Forecasting distress in european sme portfolios	Journal of banking and finance, 64, 112–135	2016	Green	1.3125	9	https://doi.org/10.1016/j.jbankfin.2015.12.007
sigrist f. (2019)	sigrist f.; hirnschall c	Grabit: gradient tree-boosted tobit models for default prediction	Journal of banking and finance, 102, 177–192	2019	Green	1.2143	1	https://doi.org/10.1016/j.jbankfin.2019.03.004
yoshino n. (2015)	yoshino n.; taghizadeh-hesary f	Analysis of credit ratings for small and medium-sized enterprises: evidence from asia	Asian development review, 32(2), 18–37	2015	Green	1.1928	11	https://doi.org/10.1162/adev_a_00050
gupta j. (2014)	gupta j.; wilson n.; gregoriou a.; healy j	The effect of internationalisation on modelling credit risk for smes: evidence from uk market	Journal of international financial markets, institutions and money, 31(1), 397–413	2014	Green	1.1290	7	https://doi.org/10.1016/j.intfin.2014.05.001
gupta j. (2014a)	gupta j.; wilson n.; gregoriou a.; healy j	The value of operating cash flow in modelling credit risk for smes	Applied financial economics, 24(9), 649–660	2014	Green	1.1290	7	https://doi.org/10.1080/09603107.2014.896979
lin s.-m. (2012)	lin s.-m.; ansell j.; andreeva g	Predicting default of a small business using different definitions of financial distress	Journal of the operational research society, 63(4), 539–548	2012	Green	1.0962	19	https://doi.org/10.1057/jors.2011.65
wu c. (2000)	wu c.; wang x.-m	A neural network approach for analyzing small business lending decisions	Review of quantitative finance and accounting, 15(3), 259–276	2000	Green	1.0000	22	https://doi.org/10.1023/a:1008324023422
wilson n. (2014)	wilson n.; altanlar a	Company failure prediction with limited information: newly incorporated companies	Journal of the operational research society, 65(2), 252–264	2014	Green	0.9677	6	https://doi.org/10.1057/jors.2013.31
pederzoli c. (2013)	pederzoli c.; thoma g.; torricelli c	Modelling credit risk for innovative smes: the role of innovation measures	Journal of financial services research, 44(1), 111–129	2013	Green	0.5063	8	https://doi.org/10.1007/s10693-012-0152-0
lugovskaya l. (2010)	lugovskaya l	Predicting default of russian smes on the basis of financial and non-financial variables	Journal of financial services marketing, 14(4), 301–313	2010	Green	0.4286	9	https://doi.org/10.1057/fsm.2009.28
kosmidis k. (2014)	kosmidis k.; stavropoulos a	Corporate failure diagnosis in smes: a longitudinal analysis based on alternative prediction models	International journal of accounting and information management, 22(1), 49–67	2014	Green	0.3226	2	https://doi.org/10.1108/ijaim-01-2013-0001
modina m. (2014)	modina m.; pietrovito f	A default prediction model for italian smes: the relevance of the capital structure	Applied financial economics, 24(23), 1537–1554	2014	Green	0.3226	2	https://doi.org/10.1080/09603107.2014.927566
abdullah n.a.h. (2016)	abdullah n.a.h.; ahmad a.h.; zainudin n.; rus r.m	Modelling small and medium-sized enterprises' failure in malaysia	International journal of entrepreneurship and small business, 28(1), 101–116	2016	Green	0.2917	2	https://doi.org/10.1504/ijesb.2016.075686
baixauli j.s. (2010)	baixauli j.s.; módica-milo a	The bias of unhealthy smes in bankruptcy prediction models	Journal of small business and enterprise development, 17(1), 60–77	2010	Green	0.2381	5	https://doi.org/10.1108/14626001011019134
yazdanfar d. (2011)	yazdanfar d	Predicting bankruptcy among smes: evidence from swedish firm-level data	International journal of entrepreneurship and small business, 14(4), 551–565	2011	Green	0.2017	3	https://doi.org/10.1504/ijesb.2011.043475
mittal s. (2011)	mittal s.; gupta p.; jain k	Neural network credit scoring model for micro enterprise financing in india	Qualitative research in financial markets, 3(3), 224–242	2011	Green	0.2017	3	https://doi.org/10.1108/17554171111176921
rikkers f. (2011)	rikkers f.; thibeault a.e	Default prediction of small and medium-sized enterprises with industry effects	International journal of banking, accounting and finance, 3(2–3), 207–231	2011	Green	0.0672	1	https://doi.org/10.1504/ijbaaf.2011.041455
habachi m. (2019)	habachi m.; benbachir s	Combination of linear discriminant analysis and expert opinion for the construction of credit rating models: the case of smes	Cogent business and management, 6(1)	2019	Green	0.0000	0	https://doi.org/10.1080/23311975.2019.1685926
gabbianelli l. (2018)	gabbianelli l	A territorial perspective of sme’s default prediction models	Studies in economics and finance, 35(4), 542–563	2018	Green	0.0000	0	https://doi.org/10.1108/sef-08-2016-0207
gupta j. (2018)	gupta j.; barzotto m.; khorasgani a	Does size matter in predicting smes failure?	International journal of finance and economics, 23(4), 571–605	2018	Green	0.0000	0	https://doi.org/10.1002/ijfe.1638
andrikopoulos p. (2018)	andrikopoulos p.; khorasgani a	Predicting unlisted smes’ default: incorporating market information on accounting-based models for improved accuracy	British accounting review, 50(5), 559–573	2018	Green	0.0000	0	https://doi.org/10.1016/j.bar.2018.02.003
altman e.i. (2018)	altman e.i.; esentato m.; sabato g	Assessing the credit worthiness of italian smes and mini-bond issuers	Global finance journal	2018	Green	0.0000	0	https://doi.org/10.1016/j.gfj.2018.09.003
ciampi f. (2018)	ciampi f.; cillo v.; fiano f	Combining kohonen maps and prior payment behavior for small enterprise default prediction	Small business economics	2018	Green	0.0000	0	https://doi.org/10.1007/s11187-018-0117-2
chai n. (2019)	chai n.; wu b.; yang w.; shi b	A multicriteria approach for modeling small enterprise credit rating: evidence from china	Emerging markets finance and trade, 55(11), 2523–2543	2019	Blue	12.1429	10	https://doi.org/10.1080/1540496x.2019.1577237
oliveira m.d.n.t. (2017)	oliveira m.d.n.t.; ferreira f.a.f.; pérez-bustamante ilander g.o.; jalali m.s	Integrating cognitive mapping and mcda for bankruptcy prediction in small-and medium-sized enterprises	Journal of the operational research society, 68(9), 985–997	2017	Blue	3.2308	7	https://doi.org/10.1057/s41274-016-0166-3
gonçalves t.s.h. (2016)	gonçalves t.s.h.; ferreira f.a.f.; jalali m.s.; meidutė-kavaliauskienė i	An idiosyncratic decision support system for credit risk analysis of small and medium-sized enterprises	Technological and economic development of economy, 22(4), 598–616	2016	Blue	1.7500	12	https://doi.org/10.3846/20294913.2015.1074125
dietsch m. (2002)	dietsch m.; petey j	The credit risk in sme loans portfolios: modeling issues, pricing, and capital requirements	Journal of banking and finance, 26(2–3), 303–322	2002	Blue	1.0000	50	https://doi.org/10.1016/s0378-4266(01)00224-2
moon t.h. (2010)	moon t.h.; sohn s.y	Technology credit scoring model considering both sme characteristics and economic conditions: the korean case	Journal of the operational research society, 61(4), 666–675	2010	Blue	0.9524	20	https://doi.org/10.1057/jors.2009.7
sohn s.y. (2013)	sohn s.y.; kim y.s	Behavioral credit scoring model for technology-based firms that considers uncertain financial ratios obtained from relationship banking	Small business economics, 41(4), 931–943	2013	Blue	0.9494	15	https://doi.org/10.1007/s11187-012-9457-5
moon t.h. (2011)	moon t.h.; kim y.; sohn s.y	Technology credit rating system for funding smes	Journal of the operational research society, 62(4), 608–615	2011	Blue	0.7395	11	https://doi.org/10.1057/jors.2010.15
sohn s.y. (2012)	sohn s.y.; doo m.k.; ju y.h	Pattern recognition for evaluator errors in a credit scoring model for technology-based smes	Journal of the operational research society, 63(8), 1051–1064	2012	Blue	0.4615	8	https://doi.org/10.1057/jors.2011.105
corazza m. (2016)	corazza m.; funari s.; gusso r	Creditworthiness evaluation of italian smes at the beginning of the 2007–2008 crisis: an mcda approach	North american journal of economics and finance, 38, 1–26	2016	Blue	0.4375	3	https://doi.org/10.1016/j.najef.2016.05.008
sohn s.y. (2010)	sohn s.y.; jeon h	Competing risk model for technology credit fund for small and medium-sized enterprises	Journal of small business management, 48(3), 378–394	2010	Blue	0.3810	8	https://doi.org/10.1111/j.1540-627x.2010.00299.x
yu s. (2019)	yu s.; chi g.; jiang x	Credit rating system for small businesses using the k-s test to select an indicator system	Management decision, 57(1), 229–247	2019	Blue	0.0000	0	https://doi.org/10.1108/md-06-2017-0553
chi g. (2019)	chi g.; yu s.; zhou y	A novel credit evaluation model based on the maximum discrimination of evaluation results	Emerging markets finance and trade	2019	Blue	0.0000	0	https://doi.org/10.1080/1540496x.2019.1643717
giannopoulos v. (2019)	giannopoulos v.; aggelopoulos e	Predicting sme loan delinquencies during recession using accounting data and sme characteristics: the case of greece	Intelligent systems in accounting, finance and management, 26(2), 71–82	2019	Blue	0.0000	0	https://doi.org/10.1002/isaf.1456
chi g. (2018)	chi g.; meng b	Debt rating model based on default identification: empirical evidence from chinese small industrial enterprises	Management decision	2018	Blue	0.0000	0	https://doi.org/10.1108/md-11-2017-1109
pan h. (2017)	pan h.; kang m.-s.; ha h.-y	Do trade area grades really affect credit ratings of small businesses? An application of big data	Management decision, 55(9), 2038–2052	2017	Blue	0.0000	0	https://doi.org/10.1108/md-11-2016-0834
altman e.i. (2007)	altman e.i.; sabato g	Modelling credit risk for smes: evidence from the u.s. Market	Abacus, 43(3), 332–357	2007	Yellow	2.2663	160	https://doi.org/10.1111/j.1467-6281.2007.00234.x
angelini e. (2008)	angelini e.; di tollo g.; roli a	A neural network approach for credit risk evaluation	Quarterly review of economics and finance, 48(4), 733–755	2008	Yellow	1.8369	107	https://doi.org/10.1016/j.qref.2007.04.001
fantazzini d. (2009)	fantazzini d.; figini s	Default forecasting for small-medium enterprises: does heterogeneity matter?	International journal of risk assessment and management, 11(1–2), 138–163	2009	Yellow	1.500	3	https://doi.org/10.1504/ijram.2009.022202
li k. (2019)	li k.; niskanen j.; niskanen m	Capital structure and firm performance in european smes: does credit risk make a difference?	Managerial finance, 45(5), 582–601	2019	Yellow	1.2143	1	https://doi.org/10.1108/mf-01-2017-0018
keasey k. (1987)	keasey k.; watson r	Non‐financial symptoms and the prediction of small company failure: a test of argenti's hypotheses	Journal of business finance & accounting, 14(3), 335–354	1987	Yellow	1.0000	134	https://doi.org/10.1111/j.1468-5957.1987.tb00099.x
keasey k. (1986)	keasey k.; watson r	The prediction of small company failure: some behavioural evidence for the uk	Accounting and business research, 17(65), 49–57	1986	Yellow	1.0000	29	https://doi.org/10.1080/00014788.1986.9729781
laitinen e.k. (1999)	laitinen e.k.; gin chong h	Early-warning system for crisis in smes: preliminary evidence from finland and the uk	Journal of small business and enterprise development, 6(1), 89–102	1999	Yellow	1.0000	23	https://doi.org/10.1108/eum0000000006665
keasey k. (1988)	keasey k.; watson r	The non-submission of accounts and small company financial failure prediction	Accounting and business research, 19(73), 47–54	1988	Yellow	1.0000	16	https://doi.org/10.1080/00014788.1988.9728835
shailer g. (1989)	shailer g	The predictability of small enterprise failures: evidence and issues	International small business journal, 7(4), 54–58	1989	Yellow	1.0000	8	https://doi.org/10.1177/026624268900700405
dasilas a. (2015)	dasilas a.; papasyriopoulos n	Corporate governance, credit ratings and the capital structure of greek sme and large listed firms	Small business economics, 45(1), 215–244	2015	Yellow	0.8675	8	https://doi.org/10.1007/s11187-015-9648-y
figini s. (2011)	figini s.; giudici p	Statistical merging of rating models	Journal of the operational research society, 62(6), 1067–1074	2011	Yellow	0.7395	11	https://doi.org/10.1057/jors.2010.41
alon i. (2015)	alon i.; boulanger m.; misati e.; madanoglu m	Are the parents to blame? Predicting franchisee failure	Competitiveness review, 25(2), 205–217	2015	Yellow	0.4337	4	https://doi.org/10.1108/cr-10-2014-0034
gharsalli m. (2019)	gharsalli m	High leverage and variance of smes performance	Journal of risk finance, 20(2), 155–175	2019	Yellow	0.0000	0	https://doi.org/10.1108/jrf-02-2018-0011
lussier r.n. (2010)	lussier r.n.; halabi c.e	A three-country comparison of the business success versus failure prediction model	Journal of small business management, 48(3), 360–377	2010	Purple	1.7619	37	https://doi.org/10.1111/j.1540-627x.2010.00298.x
halabí c.e. (2014)	halabí c.e.; lussier r.n	A model for predicting small firm performance: increasing the probability of entrepreneurial success in chile	Journal of small business and enterprise development, 21(1), 4–25	2014	Purple	1.2903	8	https://doi.org/10.1108/jsbed-10-2013-0141
lussier r.n. (2001)	lussier r.n.; pfeifer s	A crossnational prediction model for business success	Journal of small business management, 39(3), 228–239	2001	Purple	0.7962	84	https://doi.org/10.1111/0447-2778.00021
gyimah p. (2019)	gyimah p.; appiah k.o.; lussier r.n	Success versus failure prediction model for small businesses in ghana	Journal of african business	2019	Purple	0.0000	0	https://doi.org/10.1080/15228916.2019.1625017
baidoun s.d. (2018)	baidoun s.d.; lussier r.n.; burbar m.; awashra s	Prediction model of business success or failure for palestinian small enterprises in the west bank	Journal of entrepreneurship in emerging economies, 10(1), 60–80	2018	Purple	0.0000	0	https://doi.org/10.1108/jeee-02-2017-0013

## Footnotes

1 New and pioneering work on applying (1) Artificial Intelligence (AI) to a more holistic scoring approach to SME credit scoring, and (2) Stress testing during the COVID-19 pandemic by credit risk model vendors, for example Wiserfunding, Ltd. (London), has expanded the traditional purely statistical methodologies and has shown to increase the prediction accuracy results (https://www.wiserfunding.com).

2 Even if it is well known that SMEs are more informationally opaque than large corporates, (Hyytinen and Pajarinen 2008) suggest that the lender-borrower information is more connected to the firm age, rather than the firm size.

3 On the effects generated by a strict firm-bank relationship, (Matias Gama and Van Auken 2015) show that a single bank relationship may generate higher cost of debt for the company.

4 The connection map shows how the Pederzoli, Thoma and Torricelli (Pederzoli et al. 2013) study is very close to the blue cluster, which is centered on the role of innovation related SME default predictors.

5 As qualitative information, they consider unstructured data from expert opinions about the payment history, the company life cycle phase (developing, stagnating or declining) and the situation regarding orders from customers (good or declining).

6 In the practitioners’ operations, there are few seminal applications which tried to integrate traditional default risk models with the use of artificial intelligence to automatically capture non-financial information, macro-economic and industry variables (see i.e. https://www.modefinance.com/en; https://www.wiserfunding.com; http://www.zest.ai/; https://www.experian.co.uk/consumer/experian-credit-score.html).
